# Supplementary material for: Chromothripsis during telomere crisis is independent of NHEJ, and consistent with a replicative origin
Source: Genome Res. 2019 May;29(5):737–49. doi: 10.1101/gr.240705.118 (PMC6499312; doi:10.1101/gr.240705.118)
Supplement: Supplemental Material [file supp_gr.240705.118_Supplemental_file_1.zip › contigs/annotated_contigs/DB50/contig.2.DB50_length_278_mean_cov_4.71942446043.docx]

**DB50_length_278_mean_cov_4.71942446043**

```
TCTGAGTCTCGCCGTTCGGGC|TTTCCTACCAATCCCGGCAGGATCACCTTCACTGGTGCTGTGAATGAAGGAAACAACTAACTTCCTT
                      >chr4:82515266-82515397 + E=1e-64 p=0e+00
TCACAAAGCATAAGAACATTTTCATTTTCTCCTTTCTCTCTCTCCCTG|TTTTTTTTTTTTTTTT|AGAGATGAGGTCTCACCATGTTG
                                                 >chr4:82515433-82515554 + E=1e-52
CCCAGACTGGAGTGCAGTGGCTGCTTTCAAACAATCCTCCCACCTCAGCCTCCTGAATGGCGGGGATTACAGGCCCAGGTCA|GAGGCC

TGGGTGTCTTTTTTT
```
